# Evolution of Intrinsic and Extrinsic Electron Traps at Grain Boundary during Sintering ZnO Based Varistor Ceramics

**DOI:** 10.3390/ma15031098

**Published:** 2022-01-30

**Authors:** Pengkang Xie, Ziyue Wang, Kangning Wu

**Affiliations:** 1 State Key Laboratory of Disaster Prevention and Reduction for Power Grid Transmission and Distribution Equipment, Changsha 410000, China; xiepengkang@126.com; 2 Northwest Electric Power Design Institute Co., Ltd. of China Power Engineering Consulting Group, Xi’an 710075, China; wangziyue@nwepdi.com; 3 State Key Laboratory of Electrical Insulation and Power Equipment, Xi’an Jiaotong University, Xi’an 710049, China

**Keywords:** zinc oxide, varistor, Schottky barrier, defects, quenching

## Abstract

In this paper, evolution of microstructures, electrical properties and defects of the double Schottky barrier during the sintering process were investigated by quenching ZnO varistor ceramics at different sintering stages. It was found that morphology of the samples changed little when the temperature was below 800 °C. Remarkable enhancement of the Schottky barrier height and electrical properties took place in the temperature range between 600 °C and 800 °C. The Bi-rich intergranular layer changed from *β* phase to *α* phase. The interfacial relaxation at depletion/intergranular layers became detectable in the samples. Meanwhile, a distinct relaxation loss peak from electron trapping of interface states was observed instead of two dispersed ones. It indicated that the differences among the Schottky barriers in ZnO varistor ceramics became smaller with the process of sintering, which was also supported by the admittance spectra. In addition, oxygen vacancy was found more sensitive to the sintering process than zinc interstitial. The results could provide guidance for fine manipulating the Schottky barrier and its underlying defect structures by optimizing sintering process.

## 1. Introduction

Electronic ceramics with variable resistance on voltages are of importance in protecting electrical and electronic devices from overvoltage [1,2,3,4,5]. Among various kinds of varistor ceramics, ZnO based varistor ceramics are the most widely employed ones, due to their extremely high nonlinear coefficient and excellent energy absorption capability [6,7]. It has been acknowledged that the unique nonlinear current-voltage characteristics of ZnO based varistor ceramics originate from the double Schottky barriers (DSBs) at grain boundaries (GBs) [5,8], which further root in the underlying defect structures including the negatively charged interface sates and the positively charged intrinsic point defects in depletion layers [9]. Either electrical nonlinearity or stability is determined by the electron trapping behaviors of those defects and their interactions [8,10,11].

Generally, the sintering process, especially the cooling process, is acknowledged to play important roles in the formation of DSBs at GBs [9,12,13,14]. Electrical properties of ZnO based varistor ceramics are largely dependent on the sintering temperature, atmosphere and cooling rates, which are further correlated with grain size distribution and height of DSBs at GBS. Therefore, a number of studies about the sintering process mainly focused on the microstructural evolution, grain growth kinetics and phase formation of ZnO based ceramics [15,16,17,18,19]. Leite et al. [18] found that the densifying process in ZnO-Bi_2_O_3_ based ceramics was controlled by the formation and decomposition of the Zn_2_Bi_3_Sb_3_O_14_ pyrochlore phase. Arefin et al. [20] investigated the phase formation during the liquid sintering of the basic ZnO-Bi_2_O_3_-Sb_2_O_3_ system. Development of interfacial microstructure during cooling of ZnO varistor ceramics were investigated by Olsson et al. [16]. They characterized the phase transition of Bi-rich intergranular layers in the samples quenched at different temperatures. The Bi-rich intergranular phase acts as a pass way for oxygen transport. DSBs were formed during the cooling process when sufficient oxygen was absorbed at GBs [16,21,22]. Currently, the microstructural evolution of ZnO varistor ceramics during the sintering process is relatively clear.

However, evolution of the DSB that is a bridge connecting the microstructure and the electrical nonlinear properties of ZnO based varistor ceramics was seldom reported. The functional DSBs are attached to the morphology of ZnO varistor ceramics, whose electrical responses rely on the electron transport across DSBs. It is commonly accepted that interface states, which originated from absorbed oxygen, zinc vacancy, etc., are formed during the cooling process [8,23]. As a result, depletion layers are formed because free electrons nearby the surface of grains are exhausted by those negatively charged interface states, leaving only positively charged immoveable cations. Intrinsic point defects like zinc interstitials and oxygen vacancies are the major ions in the depletion layers [24,25]. Either the short-term electrical properties or long-term degradation is inevitably modulated by electron trapping behaviors of interface states and intrinsic point defects in ZnO varistor ceramics [10,23,26,27,28]. Generally, a high barrier height is related with a high donor density in depletion layers. A low density of zinc interstitials is beneficial for the anti-degradation properties of ZnO varistor ceramics. Unfortunately, evolution of the defect structures, especially those of extrinsic defects, of ZnO varistor ceramics during the sintering process is still not clear.

In this paper, ZnO varistor ceramic samples were quickly quenched at different sintering stages. In addition to the evolution of microstructures and overall electrical properties, dielectric relaxations originated from electron trapping behaviors of point defects at GBs were characterized to demonstrate the evolution of defect structures of DSBs. Relationships among microstructures, defects, DSBs and electrical properties were further discussed.

## 2. Materials and Methods

ZnO-Bi_2_O_3_ based varistor ceramics with a commercial formula were prepared by the traditional solid-state reaction method in this paper. The raw materials were mixed and ball milled in a polyamides bottle for 12 h with a speed of 300 r/min. Then, the mixture was dried, sieved and calcined at 600 °C for 3 h. The calcined powders were mixed with 1 wt% polyvinyl alcohol (PVA) solution as a binder and pressed into discs of 12 mm in diameter and 2 mm in thickness. Subsequently, they were slowly heated to 450 °C for 4 h with a heating rate of 50 °C/h to remove the PVA and residual moisture. Finally, the green discs were sintered in air.

For those as-prepared samples, they were sintered at 1200 °C for 2 h with a heating rate of 150 °C/h and a cooling rate of 100 °C/h. For those quenched samples, the sintering process, which was identical to that of the as-prepared samples, was interrupted by quickly placing them on a stainless block in air. Samples were quenched from 1200 (including two kinds of samples sintered for 1 h and 2 h, respectively), 1000, 800, 600 and 400 °C to room temperature in 3 min (labeled as Q-1200-1h, Q-1200-2h, Q-1000, Q-800, Q-600, Q-400), respectively.

A scanning electron microscope (SEM, VE-9800, Keyence, Japan) was used to characterize the surface morphologies of the samples and X-ray diffraction (XRD, D8 Advances, Bruker, German) was employed to measure the phase compositions. Gold electrodes were prepared on both sides of the samples by using a sputter coater (KYKY SBC-128, Shanghai, China) for electrical measurements. Current-voltage (*J−E*) characteristics were measured by using a DC power source (WJ10001D, Chengdu, China) and a multi-function digital meter (HP34401A, Palo Alto, Cal., USA) at room temperature. Dielectric properties were measured by a broadband dielectric measurement system (Novocontrol, Concept 80, Frankfurt, Germany) in the temperature range from −140 to 220 °C and the frequency range from 10^−1^ to 10^7^ Hz.

## 3. Results and Discussions

### 3.1. Surface Morphology and Phase Composition

**Figure 1:**
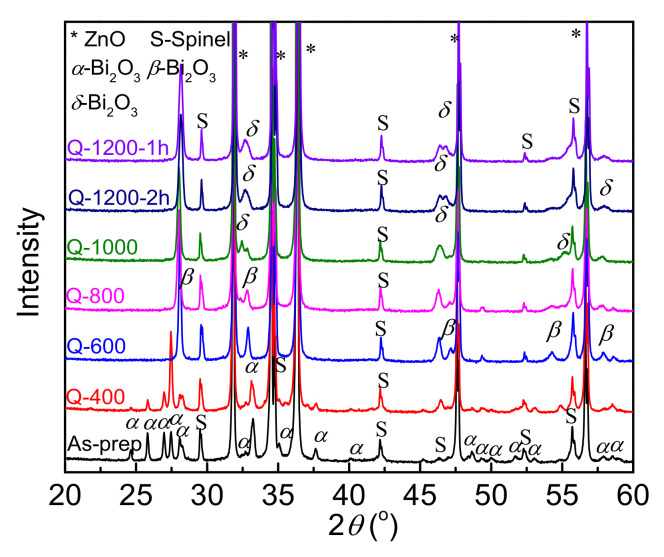
XRD patterns of ZnO ceramic samples quenched at different temperatures.

XRD patterns of the samples are presented in Figure 1, which clearly demonstrates that ZnO phase (JCPDS card no. 79-0206) is the main phase. In addition, some impurities of Bi-rich phases and spinel phases (JCPDS card no. 82-1101) are detectable, as well. In accordance with other previous reports [16,22], the Bi-rich phases are different in samples quenched at different temperatures. In the samples with elevated quenching temperatures, i.e., samples Q-1200-2h and Q-1000, the Bi-rich phase is mainly observed as *δ*−Bi_2_O_3_ cubic phase (JCPDS card no. 42-0185). In the samples Q-800 and Q-600, it is found to be *β*−Bi_2_O_3_ tetragonal phase (JCPDS card no. 74-1374). When the quenching temperature is further lowered below 600 °C, *α*−Bi_2_O_3_ phase (JCPDS card no. 71-2274) is observed in both Q-400 and as-prepared samples.

**Figure 2:**
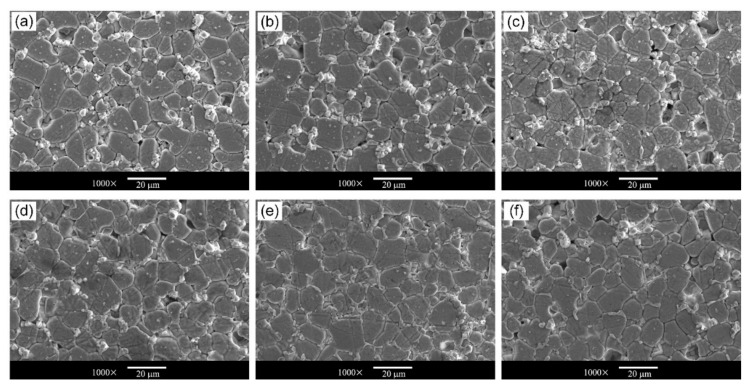
SEM pictures of ZnO ceramic samples of Q-1200-2h (**a**), Q-1000 (**b**), Q-800 (**c**), Q-600 (**d**), Q-400 (**e**) and as-prepared samples (**f**).

**Figure 3:**
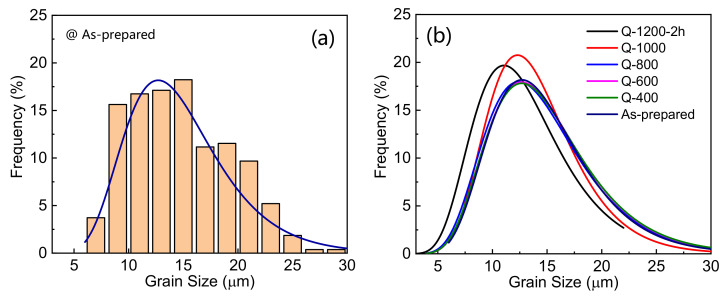
Statistic results of grain size distribution of the as-prepared ZnO ceramic sample (**a**) and fitting curves of grain size distribution of all the samples based on long-normal distributions (**b**).

In order to investigate the evolution of grain size distribution, polished surface morphologies of samples are exhibited in Figure 2. Uniform grains are formed in all the studied samples. In addition, some intergranular phases and spinel phases mainly distribute at junctions of grains, which is similar to other reports [29]. Grain size distribution is further statistically measured [30]. More than 200 grains were measured for each sample. The grains were distinguished after image binarization by the software ImageJ [31]. Subsequently, areas of grains were calculated and the equivalent grain sizes were obtained. Taking the as-prepared ZnO varistor ceramics as an example, grain size distribution is plotted in Figure 3a, which fits well with the log-normal distribution [29,30,32]:

(1) $$g\left(x\right)=\frac{1}{\sqrt{2\pi }x\sigma }\times \exp\left(\frac{-{\left(\ln x-\mu \right)}^{2}}{2\sigma^{2}}\right)$$

where *g*(*x*) is the log-normal probability density function, *x* is the grain size, *μ* is the logarithmic mean and *σ* is the logarithmic standard deviation. Therefore, it can be further calculated that

(2) $$E\left(X\right)=e^{\mu +\sigma^{2}/2}$$

(3) $$D\left(X\right)=\sqrt{e^{\sigma^{2}}-1}\left(e^{\mu +\sigma^{2}/2}\right)$$

(4) $$\varepsilon =\frac{D\left(X\right)}{E\left(X\right)}=\sqrt{e^{\sigma^{2}}-1}$$

where *E*(*X*) is the mean value of the lognormal distribution, which represents the average grain size, *D*(*X*) is the standard deviation of the lognormal distribution and *ε* is the coefficient of nonuniformity.

Grain size distributions of all the samples are further plotted in Figure 3b and the corresponding parameters are listed in Table 1. With a decrease in quenching temperature, the curves in Figure 3b slightly move towards larger grain size. As a result, the average grain size *E*(*X*) gradually increases from 13.23 µm for sample Q-1200-2h to 14.81 µm for the as-prepared sample. Meanwhile, the coefficient of nonuniformity *ε* decreases firstly, rises, and finally reaches to a constant. It indicates that grains trend to be more uniform in the temperature range from 1200 to 1000 °C, in which small grains disappear. Subsequently, slight increases in the sizes of those large grains leads to a slight increase in the coefficient of nonuniformity *ε* in the temperature range from 1000 to 800 °C. The fitting curves in Figure 3b are almost coincided with each other, when the quenching temperature is blow 800 °C. It indicates the grains of the ZnO varistor ceramics would grow little as long as the temperature was below 800 °C.

### 3.2. Nonlinear Current-Voltage Characteristics

**Figure 4:**
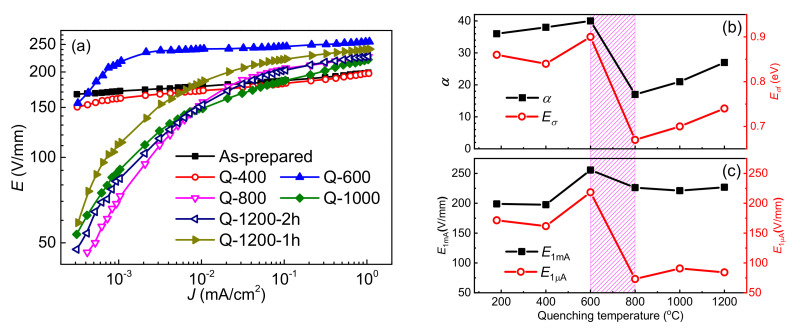
The *J-E* curves of ZnO ceramic samples quenched at different temperatures (**a**). Dependence of nonlinear coefficient *α* and activation energy of conductivity *E_σ_*_λ_ (**b**) and electric fields at 1 mA/cm^2^ and 1 μA/cm^2^, i.e., *E*_1mA_ and *E*_1μA_, (**c**) on the quenching temperature.

Figure 4a demonstrates the nonlinear current-voltage characteristics of the samples at room temperature. Electrical parameters in detail are listed in Table 2, including electric fields at 1 mA/cm^2^ and 1 µA/cm^2^ (*E*_1mA_ and *E*_1µA_), and nonlinear coefficient *α*. Noticeably, remarkable discrepancies in current-voltage characteristics are observed with 600–800 °C as a critical temperature range. A remarkable increase in nonlinear coefficient *α* from ∼20 to ∼40 is observed in Figure 4b, when the quenching temperature decreases from 800 to 600 °C. Similarly, an abrupt increase in *E*_1µA_, as shown in Figure 4c, is observed in this temperature range. As a comparison, the *E*_1mA_ varies little on the quenching temperature, which is a characteristic of the formation of DSB in ZnO varistor ceramics [8,33]. It is thus reasonable to deduce that well developed DSBs are mainly formed in this temperature range between 600 and 800 °C.

### 3.3. Evolution of Intrinsic Point Defects in Depletion Layers

**Figure 5:**
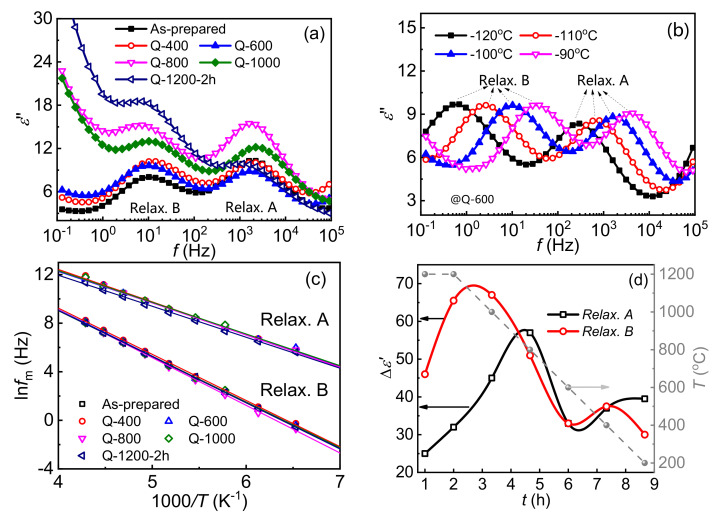
*ε*″-spectra ZnO samples at low temperatures. (**a**) *ε*″-spectra of the ZnO samples at −100 °C. (**b**) Effects of temperature on *ε*″-spectra of Q-600 samples. (**c**) Arrhenius plots of peak frequencies of relaxations A and B on the measuring temperatures. (**d**) Time delay of magnitudes of relaxations A and B with the solid gray circles as the cooling temperatures.

Electron trapping of point defects of DSBs could be presented as dielectric relaxations, so that *ε*″ spectra of samples are plotted in Figure 5a. The *ε*″ is the imaginary part of complex permittivity and dielectric relaxation could be presented as loss peaks in the frequency-domain *ε*″ spectroscopy [24]. There are two relaxations A and B at −100 °C, which correspond to intrinsic point defects in depletion layers of DSBs [24,34]. The dependences of those relaxation processes on temperature, taking the Q-600 samples as an example, is further demonstrated in Figure 5b. Both of them are found to shift towards a higher frequency range with the increase in temperature. The peak frequencies as a function of the measuring temperature fit well with the Arrhenius equation, which is presented in Figure 5c. The corresponding activation energies of relaxations A and B are listed in Table 3, which are constant to 0.23 and 0.33 eV, respectively. They are equal to the trap depth of intrinsic point defects Zn_i_¨ and V_O_˙, respectively [24]. In consequence, the evolution of them could be possibly characterized via fitting the *ε*″ spectra by using the Cole–Cole equation [23,24,35,36]:

(5) $$\varepsilon^{\prime \prime }\left(\omega \right)=k_{0}\omega^{\alpha_{0}}+\overset{n}{\underset{i=1}{\sum }}\frac{k_{i}\left(\omega \tau_{i}{)}^{1-\alpha_{i}}\cos(\pi \alpha_{i}/2\right)}{1+2\left(\omega \tau_{i}{)}^{1-\alpha_{i}}\sin(\omega \tau_{i}/2\right)+{\left(\omega \tau_{i}\right)}^{2\left(1-\alpha_{i}\right)}}$$

where *k*_0_ represents the magnitude of DC conductivity while *k_i_* (*i* = 1, 2, 3 ...) is the magnitude of permittivity contributed by the *i*th relaxation (∆*ε*_0_). *τ_i_* is the relaxation time and *α_i_* is the depression angle.

The fitted magnitudes of relaxations A and B, which equate with the densities of intrinsic point defects [37], are shown in Figure 5d. With the ongoing cooling process, a decline after a first increase in the densities of both Zn_i_¨ and V_O_˙ is observed. It should be mentioned that V_O_˙·is more sensitive to the sintering process than Zn_i_¨ because the density of Zn_i_¨ varies after that of V_O_˙. Maximum densities of Zn_i_¨ and V_O_˙·appear at about 1100 °C and 850 °C, respectively. Oxygen is lost from the lattice at sintering temperatures, so that oxygen vacancies are accumulated and zinc atoms are consequently dragged into interstitial sites. With the decrease in temperature, absorption of oxygen at GBs becomes dominant, leading to a reduction in both densities of Zn_i_¨ and V_O_˙. Their densities finally tend to be constant when the temperature is below 500 °C. It is generally acknowledged that zinc interstitials are crucial for the stability of ZnO based varistor ceramics [10,26,38]. The findings in this paper that oxygen vacancies are more sensitive might provide guidance to manipulate intrinsic point defects.

### 3.4. Evolution of Extrinsic Interfacial Defects

In addition to intrinsic point defects, extrinsic defects are also important for modulating carrier transport in ZnO based varistor ceramics. Unfortunately, their relaxation times are commonly so long that the corresponding dielectric relaxations can only be detected in low-frequency ranges. Component of DC conduction is usually intense in this region and might cover the relaxation component in those commonly used dielectric spectra, such as modulus, impedance and admittance spectra. Investigations on those electron trapping behaviors of interfacial defects are therefore hard to carry out [24,39]. In this paper, the (∂*ε′*/∂ln*ω*)/*ε′* spectroscopy, which is free of DC conduction, is employed to release dielectric relaxations from the covering of DC conduction [24,28,40,41,42,43], leaving only relaxation related loss peaks.

**Figure 6:**
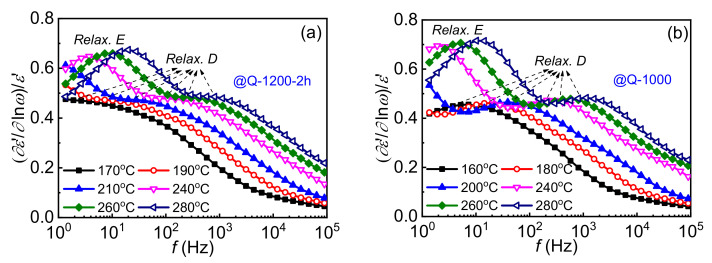

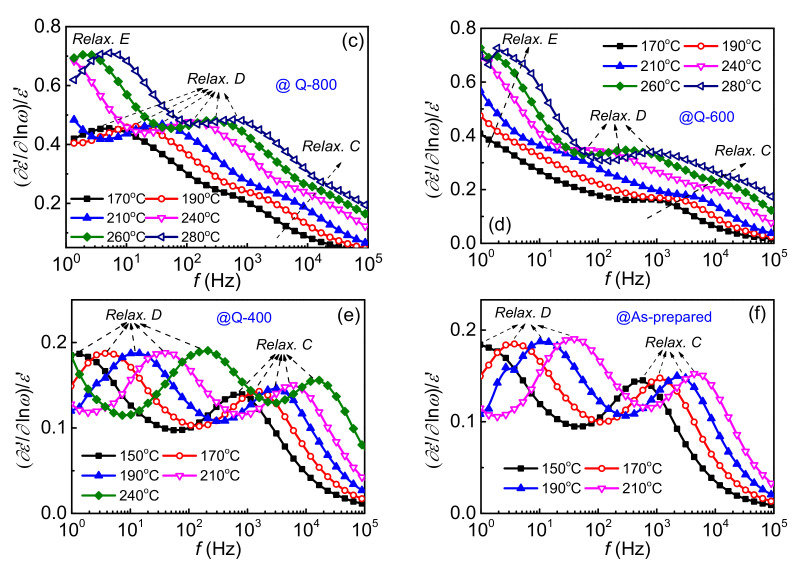
Dielectric spectra (∂*ε′*/∂ln*ω*)/*ε′* at elevated temperatures of Q-1200-2h (**a**), Q-1000 (**b**), Q-800 (**c**), Q-600 (**d**), Q-400 (**e**) and as-prepared samples (**f**).

Figure 6 shows (∂*ε′*/∂ln*ω*)/*ε′* spectra at elevated temperatures of the samples, in which three loss peaks (labeled as C, D and E with the increase in relaxation time) are observed. All the peaks follow the Arrhenius relation so that their relaxation activation energies can be calculated, which are listed in Table 3. Relaxations C and D were reported previously [24], while relaxation E is firstly found in this work.

Relaxation C is reported to be an interfacial polarization between the depletion layers of DSBs and intergranular phases [9,24,42,44], whose relaxation activation energy is similar to the activation energy of intergranular layer resistance. Therefore, relaxation C can be regarded as an indicator for the intergranular phase in ZnO based varistor ceramics. As observed in Figure 6, relaxation C is detectable unless the quenching temperature is equal to or less than 800 °C. It is only slightly observed in sample Q-800, while relatively distinct loss peaks of relaxation C could be detected in Q-600, Q-400 and as-prepared samples. In other words, relaxation C becomes detectable in the quenching temperature range from 800 to 600 °C. At about 600 °C, the intergranular phase also changes from *β*-phase into *α*-phase, as shown in Figure 1. Significantly enhanced electrical nonlinearity of the samples shown in Figure 4 also appears at this period, which is exhibited as improved *α* and reduced *E*_1μA_. The results further support that 600–800 °C is a crucial temperature range for the barrier formation of ZnO based varistor ceramics.

Similar to the relaxation C, relaxations D and E also vary greatly in this temperature range. As presented in Figure 6, relaxation E disappeared when the quenching temperature is below 600 °C. In the meantime, distinct loss peaks of relaxation D are observed instead of dispersed ones. It is notable that activation energies of relaxations D and E reach up to 0.9~1.5 eV, as listed in Table 3. The energies are even higher than the height of DSB, which is similar to the activation energy of DC conduction. It is much higher than the energy for electron trapping of intrinsic point defects and even ion migration of zinc interstitial [10]. The most possible origination for them could only be electron trapping of interface states [8].

**Figure 7:**
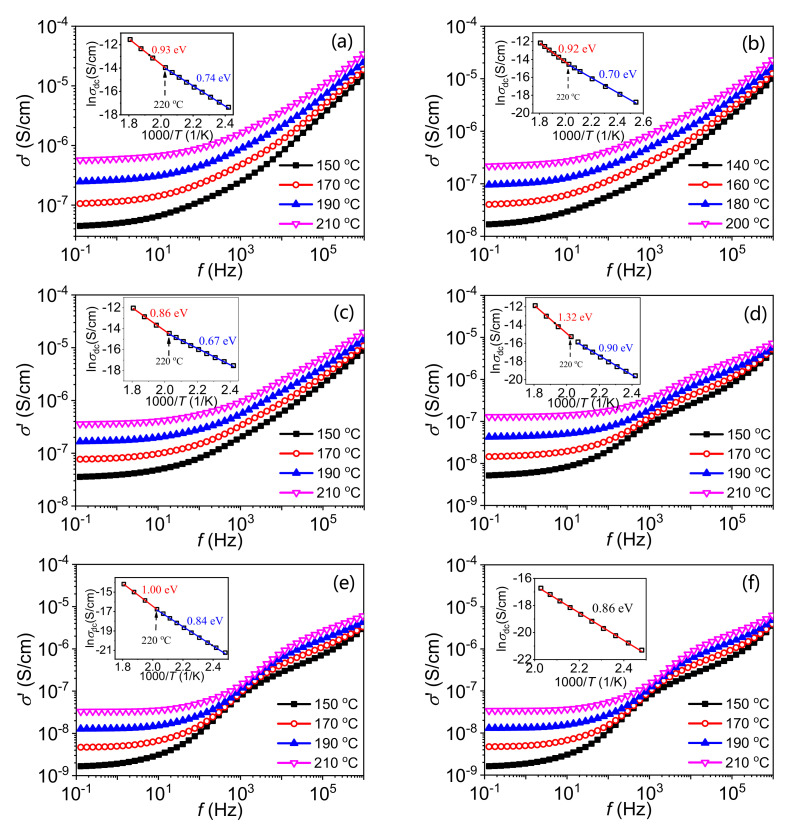
Admittance spectra of the Q-1200-2h (**a**), Q-1000 (**b**), Q-800 (**c**), Q-600 (**d**), Q-400 (**e**) and as-prepared samples (**f**). Insets are activation energies calculated by the Arrhenius relation between DC conductivities and the measuring temperatures.

Relaxation D was reported to correlate with the electronic relaxation of interface states at GBs, whose activation energy is the corresponding trap depth [8,23,24]. The appearance of the relaxation E indicates the formation of two kinds of Schottky barriers during the cooling process. Therefore, admittance spectra of the ZnO varistor ceramic samples are also plotted in Figure 7. There are two activation energies (labeled as *E_σ_*_l_ and *E_σ_*_h_) of DC conductivity, which supports the existence of two kinds of DSBs at grain boundaries. Dependence of *E_σ_*_l_ on quenching temperature is also plotted in Figure 4b as an example, which is consistent with the variation of the nonlinear coefficient. Generally, the height of DSB is pinned by interface states [8,23]. Similar to relaxation D, relaxation E might correlate with interface states. When the temperature continuously decreases during the cooling process, the difference between two kinds of DSBs gradually becomes smaller and smaller. On the one hand, relaxation E disappeared and only relaxation D is detected in the as-prepared samples. On the other hand, the *E_σ_*_h_ gradually trends to *E_σ_*_l_. Finally, only one activation energy of DC conductivity is detected in the as-prepared samples, indicating identical DSBs.

## 4. Conclusions

In conclusion, ZnO varistor ceramic samples were quenched at different sintering stages to study the evolution of microstructures, electrical properties as well as Schottky barrier and its relating intrinsic and extrinsic point defects. The quenching temperature range between 600 and 800 °C was found to be crucial to ZnO varistor ceramics. When the samples were cooled to this temperature range, phase transition of Bi-rich phase (from *β* to *α*) took place. Differences among the DSBs in a sample, which were related with the interface sates, gradually disappeared with the ongoing cooling process. Finally, uniform barriers were formed in the as-prepared samples, which was exhibited as greatly enhanced nonlinear current-voltage characteristics. In addition, oxygen vacancies were found to be more sensitive to the sintering process than zinc interstitials because zinc interstitials varied after oxygen vacancies. The findings will be of assistance to manipulation of the defects and Schottky barriers at grain boundaries of ZnO varistor ceramics. A high barrier height required sufficient donor point defects in depletion layers, while the anti-degradation performance was negatively correlated with the densities of zinc interstitials. A fine manipulation on point defects was vital to the synergic optimization of both short-term and long-term properties of ZnO varistor ceramics.

## Figures and Tables

**Table 1:** Average grain sizes for the quenched and as-prepared samples.

Samples	Q-1200-2h	Q-1000	Q-800	Q-600	Q-400	As-Prepared
*μ*	2.52	2.60	2.63	2.65	2.65	2.64
*σ*	0.34	0.30	0.34	0.33	0.33	0.32
*E*(*X*) (µm)	13.23	14.06	14.73	14.96	14.99	14.81
*D*(*X*) (µm)	4.71	4.30	5.12	5.11	5.15	4.86
*ε* (%)	35.60	30.58	34.77	34.16	34.36	32.84

**Table 2:** Electrical parameters of the quenched and as-prepared ZnO ceramic samples.

Samples	Q-1200-1h	Q-1200-2h	Q-1000	Q-800	Q-600	Q-400	As-Prepared
*α*	28.0	27.5	21.3	17.1	40.6	38.3	36.4
*E*_1mA_ (V∙cm^−1^)	240.7	226.8	221.1	226.1	255.6	197.6	198.9
*E*_1μA_ (V∙cm^−1^)	112.9	84.2	90.7	73.1	218.4	161.6	171.4

**Table 3:** Activation energies of dielectric relaxations and DC conduction of samples.

Samples	*E*_A_/eV	*E*_B_/eV	*E*_C_/eV	*E*_D_/eV	*E*_E_/eV	*E_σσ_*/eV	
						*E_σ_*_l_	*E_σ_*_h_
Q-1200-2h	0.22	0.32	--	1.12	1.52	0.74	0.93
Q-1000	0.23	0.33	--	0.89	1.01	0.70	0.92
Q-800	0.23	0.33	--	0.89	1.08	0.67	0.86
Q-600	0.22	0.33	0.60	1.44	--	0.90	1.32
Q-400	0.23	0.32	0.62	1.05	--	0.84	1.00
As-prepared	0.23	0.32	0.63	1.08	--	0.86	

## Data Availability

Not applicable.

## References

[1] Greuter F. (2021). Zno varistors: From grain boundaries to power applications. Oxide Electronics.

[2] Tang Z., Ning K., Fu Z., Lian Z., Wu K., Huang S. (2022). Significantly enhanced varistor properties of CaCu_3_Ti_4_O_12_ based ceramics by designing superior grain boundary: Deepening and broadening interface states. J. Mater. Sci. Technol..

[3] Tian T., Zheng L., Podlogar M., Zeng H., Bernik S., Xu K., Ruan X., Shi X., Li G. (2021). Novel ultrahigh-performance ZnO-based varistor ceramics. ACS Appl. Mater. Inter..

[4] Tabasco-Novelo C., Cervantes-López J.L., González-Panzo I.J., Rodríguez-Gattorno G., Quintana P. (2021). High non-linear electrical properties of Li_3x_Co_7__-4x_Sb_2+x_O_12_ a new ceramic varistor. J. Alloys Compd..

[5] He J. (2019). Metal Oxide Varistors.

[6] Zhang C., Xing H., Li P., Li C., Lv D., Yang S. (2019). An experimental study of the failure mode of ZnO varistors under multiple lightning strokes. Electronics.

[7] Guo M., Zhao X., Shi W., Zhang B., Wu K., Li J. (2022). Simultaneously improving the electrical properties and long-term stability of ZnO varistor ceramics by reversely manipulating intrinsic point defects. J. Eur. Ceram. Soc..

[8] Blatter G., Greuter F. (1986). Carrier transport through grain boundaries in semiconductors. Phys. Rev. B.

[9] Guo M., Wang Y., Wu K., Zhang L., Zhao X., Lin Y., Li J. (2020). Revisiting the effects of Co_2_O_3_ on multiscale defect structures and relevant electrical properties in ZnO varistors. High Volt..

[10] He J., Cheng C., Hu J. (2016). Electrical degradation of double-schottky barrier in ZnO varistors. AIP Adv..

[11] Meng P., Zhao X., Fu Z., Wu J., Hu J., He J. (2019). Novel zinc-oxide varistor with superior performance in voltage gradient and aging stability for surge arrester. J. Alloys Compd..

[12] Wang H., Zhao H., Liang W., Fan S., Kang J. (2021). Effect of sintering process on the electrical properties and microstructure of Ca-doped ZnO varistor ceramics. Mat. Sci. Semicon. Proc..

[13] Roy S., Das D., Roy T.K. (2018). Influence of sintering temperature on microstructure and electrical properties of Er_2_O_3_ added ZnO-V_2_O_5_-MnO_2_-Nb_2_O_5_ varistor ceramics. J. Alloys Compd..

[14] Gunnewiek R.F.K., Kiminami R.H.G.A. (2017). Two-step microwave sintering of nanostructured ZnO-based varistors. Ceram. Int..

[15] Peiteado M., De La Rubia M.A., Fernández J.F., Caballero A.C. (2006). Thermal evolution of ZnO-Bi_2_O_3_-Sb_2_O_3_ system in the region of interest for varistors. J. Mater. Sci..

[16] Olsson E., Dunlop G.L., Österlund R. (1989). Development of interfacial microstructure during cooling of a ZnO varistor material. J. Appl. Phys..

[17] Roy S., Roy T.K., Das D. (2019). Grain growth kinetics of Er_2_O_3_ doped ZnO-V_2_O_5_ based varistor ceramics. Ceram. Int..

[18] Leite E.R., Nobre M.A.L., Longo E., Varela J.A. (1996). Microstructural development of ZnO varistor during reactive liquid phase sintering. J. Mater. Sci..

[19] Hembram K., Rao T.N., Srinivasa R.S., Kulkarni A.R. (2021). Cao doped ZnO-Bi_2_O_3_ varistors: Grain growth mechanism, structure and electrical properties. Ceram. Int..

[20] Arefin M.L., Raether F., Dolejš D., Klimera A. (2009). Phase formation during liquid phase sintering of ZnO ceramics. Ceram. Int..

[21] Stucki F., Greuter F. (1990). Key role of oxygen at zinc oxide varistor grain boundaries. Appl. Phys. Lett..

[22] Li J., Li S., Cheng P., Alim M.A. (2015). Advances in ZnO-Bi_2_O_3_ based varistors. J. Mater. Sci. Mater. Electron..

[23] Wu K., Wang Y., Hou Z., Li S., Li J., Tang Z., Lin Y. (2021). Colossal permittivity due to electron trapping behaviors at the edge of double Schottky barrier. J. Phys. D Appl. Phys..

[24] Huang Y., Wu K., Xing Z., Zhang C., Hu X., Guo P., Zhang J., Li J. (2019). Understanding the validity of impedance and modulus spectroscopy on exploring electrical heterogeneity in dielectric ceramics. J. Appl. Phys..

[25] Janotti A., Van de Walle C.G. (2007). Native point defects in ZnO. Phys. Rev. B.

[26] Gupta T.K., Carlson W.G. (1985). A grain-boundary defect model for instability/stability of a ZnO varistor. J. Mater. Sci..

[27] Wang X., Ren X., Li Z., You W., Jiang H., Yu W., Jin L., Yao Z., Shi L. (2021). A unique tuning effect of Mg on grain boundaries and grains of ZnO varistor ceramics. J. Eur. Ceram. Soc..

[28] Huang Y., Guo M., Li J. (2020). Multiscale defect responses in understanding degradation in zinc oxide varistor ceramics. Ceram. Int..

[29] Liu W., Zhang L., Kong F., Wu K., Li S., Li J. (2020). Enhanced voltage gradient and energy absorption capability in ZnO varistor ceramics by using nano-sized ZnO powders. J. Alloys Compd..

[30] Gerlt A.R.C., Criner A.K., Semiatin L., Payton E.J. (2019). On the grain size proportionality constants calculated in M.I. Mendelson’s “average grain size in polycrystalline ceramics”. J. Am. Ceram. Soc..

[31] Grzebielucka E.C., Leandro Monteiro J.F.H., de Souza E.C.F., Ferreira Borges C.P., de Andrade A.V.C., Cordoncillo E., Beltrán-Mir H., Antunes S.R.M. (2020). Improvement in varistor properties of CaCu_3_Ti_4_O_12_ ceramics by chromium addition. J. Mater. Sci. Technol..

[32] Wang Y., Hou Z., Li J., Wu K., Song J., Chen R., Li K., Hao L., Xu C. (2021). Simultaneously enhanced potential gradient and nonlinearity of ZnO varistor ceramics by MnO doping with nano-sized ZnO powders. Materials.

[33] Eda K. (1989). Zinc oxide varistors. IEEE Electr. Insul. Mag..

[34] Han J., Senos A.M.R., Mantas P.Q. (2002). Deep donors in polycrystalline Mn-doped ZnO. Mater. Chem. Phys..

[35] Ben Belgacem R., Chaari M., Braña A.F., Garcia B.J., Matoussi A. (2017). Structural, electric modulus and complex impedance analysis of ZnO/TiO2 composite ceramics. J. Am. Ceram. Soc..

[36] Miranda G.G., Lucas De Sousa E Silva R., Banerjee P., Franco A. (2020). Role of Ga presence into the heterojunction of metal oxide semiconductor on the stability and tunability ZnO ceramics. Ceram. Int..

[37] Lin J., Li S., He J., Zhang L., Liu W., Li J. (2017). Zinc interstitial as a universal microscopic origin for the electrical degradation of ZnO-based varistors under the combined dc and temperature condition. J. Eur. Ceram. Soc..

[38] Zhao H., Hu J., Chen S., Xie Q., He J. (2016). Improving age stability and energy absorption capabilities of ZnO varistors ceramics. Ceram. Int..

[39] Cheng P., Song J., Wang Q., Li S., Li J., Wu K. (2018). Fine representation of dielectric properties by impedance spectroscopy. J. Alloys Compd..

[40] Tang Z., Wu K., Li J., Huang S. (2020). Optimized dual-function varistor-capacitor ceramics of core-shell structured xBi_2/3_Cu_3_Ti_4_O_12_/(1-x)CaCu_3_Ti_4_O_12_ composites. J. Eur. Ceram. Soc..

[41] Wu K., Huang Y., Li J., Li S. (2017). Space charge polarization modulated instability of low frequency permittivity in CaCu_3_Ti_4_O_12_ ceramics. Appl. Phys. Lett..

[42] West A.R., Andres-Verges M. (1997). Impedance and modulus spectroscopy of ZnO varistors. J. Electroceram..

[43] Wu K., Huang Y., Hou L., Tang Z., Li J., Li S. (2018). Effects of dc bias on dielectric relaxations in CaCu_3_Ti_4_O_12_ ceramics. J. Mater. Sci. Mater. Electron..

[44] Zhao X., Li J., Li H., Li S. (2012). Intrinsic and extrinsic defect relaxation behavior of ZnO ceramics. J. Appl. Phys..

